# MEGAN-LR: new algorithms allow accurate binning and easy interactive exploration of metagenomic long reads and contigs

**DOI:** 10.1186/s13062-018-0208-7

**Published:** 2018-04-20

**Authors:** Daniel H. Huson, Benjamin Albrecht, Caner Bağcı, Irina Bessarab, Anna Górska, Dino Jolic, Rohan B. H. Williams

**Affiliations:** 1 Center for Bioinformatics, University of Tübingen, Sand 14, Tübingen, 72076 Germany; 2 Life Sciences Institute, National University of Singapore, 28 Medical Drive, Singapore, 117456 Singapore; 3 Max-Planck Institute for Developmental Biology, Tübingen, 72076 Germany; 4 Singapore Centre for Environmental Life Sciences Engineering, National University of Singapore, 28 Medical Drive, Singapore, 117456 Singapore; 5 IMPRS ‘From Molecules to Organisms’, Tübingen, Germany

**Keywords:** Microbiome, Long reads, Sequence analysis, Taxonomic binning, Functional binning, Algorithms, Software, Nanopore, PacBio

## Abstract

**Background:**

There are numerous computational tools for taxonomic or functional analysis of microbiome samples, optimized to run on hundreds of millions of short, high quality sequencing reads. Programs such as MEGAN allow the user to interactively navigate these large datasets. Long read sequencing technologies continue to improve and produce increasing numbers of longer reads (of varying lengths in the range of 10k-1M bps, say), but of low quality. There is an increasing interest in using long reads in microbiome sequencing, and there is a need to adapt short read tools to long read datasets.

**Methods:**

We describe a new LCA-based algorithm for taxonomic binning, and an interval-tree based algorithm for functional binning, that are explicitly designed for long reads and assembled contigs. We provide a new interactive tool for investigating the alignment of long reads against reference sequences. For taxonomic and functional binning, we propose to use LAST to compare long reads against the NCBI-nr protein reference database so as to obtain frame-shift aware alignments, and then to process the results using our new methods.

**Results:**

All presented methods are implemented in the open source edition of MEGAN, and we refer to this new extension as MEGAN-LR (MEGAN long read). We evaluate the LAST+MEGAN-LR approach in a simulation study, and on a number of mock community datasets consisting of Nanopore reads, PacBio reads and assembled PacBio reads. We also illustrate the practical application on a Nanopore dataset that we sequenced from an anammox bio-rector community.

**Reviewers:**

This article was reviewed by Nicola Segata together with Moreno Zolfo, Pete James Lockhart and Serghei Mangul.

**Conclusion:**

This work extends the applicability of the widely-used metagenomic analysis software MEGAN to long reads. Our study suggests that the presented LAST+MEGAN-LR pipeline is sufficiently fast and accurate.

## Background

There are numerous computational tools for taxonomic or functional binning or profiling of microbiome samples, optimized to run on hundreds of millions of short, high quality sequencing reads [1–4]. Alignment-based taxonomic binning of reads is often performed using the naïve LCA algorithm [5], because it is fast and its results are easy to interpret. Functional binning of reads usually involves a best-hit strategy to assign reads to functional classes.

Software or websites for analyzing microbiome shotgun sequencing samples usually provide some level of interactivity, such as MG-RAST [2]. The interactive microbiome analysis tool MEGAN, which was first used in 2006 [6], is explicitly designed to enable users to interactively explore large numbers of microbiome samples containing hundreds of millions of short reads [1].

Illumina HiSeq and MiSeq sequencers allow researchers to generate sequencing data on a huge scale, so as to analyze many samples at a great sequencing depth [7–9]. A wide range of questions, in particular involving the presence or absence of particular organisms or genes in a sample, can be answered using such data. However, there are interesting problems that are not easily resolved using short reads. For example, it is often very difficult to determine whether two genes that are detected in the same microbiome sample also belong to the same *genome*, even if they are located close to each other in the genome, despite the use of metagenomic assembly in combination with contig binning techniques and paired-end reads [10].

Current long read sequencing technologies, such as provided by Oxford Nanopore Technologies (ONT) or Pacific Biosciences (PacBio), produce smaller numbers (in the range of hundreds of thousands) of longer reads (of varying lengths in the range of 10 kb – 300 kb, say) of lower quality (error rates around 10%) [11, 12]. There is increasing interest in using long reads in microbiome sequencing and there is a need to adapt short read tools to long read datasets. There are a number of tools that are applicable to long reads, such as WIMP [13], Centrifuge [14] or Kaiju [15]. While the two former are based on comparing against DNA references, the latter can also use a protein reference database.

In this paper, we focus on protein-alignment-based approaches. One reason for this is that existing DNA reference databases cover only a small fraction of the genome sequences believed to be present in the environment [16], although much work has been done on sequencing human-associated microbes [17]. This problem can be ameliorated, to a degree, by using protein alignments, because amino acid sequences are more conserved than DNA sequences. Moreover, work on bacterial pangenomes suggest that the association between species level taxonomic assignment and coding gene content can be weak [18]. Finally, questions going beyond taxonomic profiling and correlation studies will usually require knowledge of the functional content.

Here we present a new classification pipeline for taxonomic and functional analysis of long reads and contigs, based on protein alignments. The pipeline, LAST+MEGAN-LR, consists of first running the alignment tool LAST and then processing the resulting DNA-to-protein alignments using new algorithms provided in MEGAN-LR. We perform a simulation study to evaluate the performance of the method in the context of the taxonomic assignment and compare it with Kaiju, one of the few other tools that use protein references. We also investigate the performance of the pipeline using mock-community datasets and illustrate its application on Nanopore reads sequenced from an anammox enrichment bio-rector.

## Methods

### Long read taxonomic binning

The naïve LCA (lowest common ancestor) algorithm is widely used for binning short reads onto the nodes of a given taxonomy (such as the NCBI taxonomy), based on alignments [5]. Consider a read *r* that has significant alignments $a_{1},\dots,a_{k}$ to reference sequences associated with taxa $t_{1},\dots,t_{k}$. The naïve LCA assigns *r* to the lowest taxonomic node that lies above the set of all nodes representing $t_{1},\dots,t_{k}$. The set of *significant* alignments is defined to consist of those alignments whose score lies close to the best score achieved for the given read, defined, say, as those that have a bit score that lies within 10% of the best bit score.

The naïve LCA algorithm is fast, easy to implement and the results are easy to interpret. When applied to protein alignments, an implicit assumption of the algorithm is that any read aligns to only one gene and so all associated taxa are “competing” for the same gene; this justifies the above definition of significant alignments. While reads that are only a few hundred base pairs long usually fulfill this assumption, longer reads or assembled contigs often overlap with more than one gene and so the naïve algorithm is not suitable for them.

To make the naïve algorithm applicable to protein alignments on a long read or contig *r*, a simple idea is to first determine “conserved genes” as regions along the read where alignments accumulate. The second step is to apply the naïve LCA to each of these regions individually. The placement of the read is finally determined using the LCA of all these gene-based LCAs. There are two problems here. First, because protein alignments around the same location can have quite different lengths, delineating different “conserved genes” can be difficult in practice. Second, because a large proportion of genes on a long read or contig may be conserved to different extents across different taxonomic groups, the placement of the read will often be to a high-level (or “unspecific”) taxon.

To address these issues, we present a new taxonomic binning for long reads that we call the *interval-union LCA* algorithm. This algorithm processes each read *r* in turn, in two steps. First, the read is partitioned into a set of intervals $v_{1},\dots, v_{m}$ that have the property that every alignment associated with *r* starts and ends at the beginning or end of some interval, respectively. In other words, a new interval starts wherever some alignment begins or ends. We say that an alignment *a*_*i*_ is *significant* on an interval *v*_*j*_, if its bit score lies within 10% (by default) of the best bit score seen for any alignment that covers *v*_*j*_. In MEGAN-LR this threshold is referred to as the topPercent parameter.

In the second step, for each taxon *t* that is associated with any of the alignments, let *I*(*t*) denote the union of all intervals for which there exists some significant alignment *a*_*i*_ associated with taxon *t*. In a post-order traversal, for each higher-rank taxonomic node *s* we compute *I*(*s*) as the union of the intervals covered by the children of *s*. In result, every node of the taxonomy is labeled by a set of intervals. Note that, during the computation of the union of interval sets, we merge any overlapping intervals into a single interval.

**Fig. 1:**
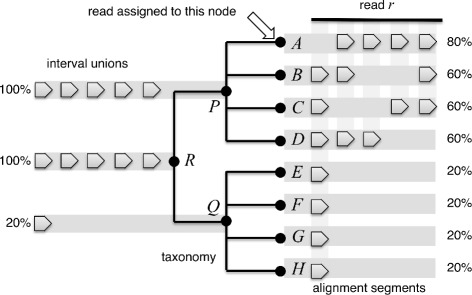
To illustrate the interval-union LCA algorithm, here we show eight hypothetical species $A,B,\dots,H$ separated into two genera, *P* and *Q*, belonging to the same family *R*. Alignments from the read *r* to proteins associated with the species are indicated by arrows on the right and cover between 80% (for *A*) and 20% (for *H*) of the aligned read. Using arrows, on the left we depict the sets of intervals computed for nodes *P*,*Q*,*R* as the union of the sets of intervals of the children of each node. Nodes *R* and *P* each cover 100% of the aligned read. The read *r* is placed on *A* as it is the lowest taxonomic node with ≥ 80*%* coverage. Note that, if *A* only covered 60% of the aligned read, then the read would be assigned to the higher taxon *P* (and this would remain the case even if one of the taxa below *Q* had 60% coverage)

The read *r* is then placed on the taxon *s* that has the property that its set of intervals *I*(*s*) covers 80% (by default) of the total aligned or covered portion of the read, while none of its children does (see Fig. 1). In MEGAN-LR this threshold is referred to as the percentToCover parameter. Note that it is possible that there are multiple nodes that have this property, in which case the read is assigned to the LCA of all such nodes.

### Long read functional binning and annotation

Functional binning of short reads is usually performed by assigning each read to a class in a functional classification system such as InterPro [19], eggNOG [20] or KEGG [21], based on its alignments.

This is often done using a simple *best-hit* strategy, as follows. For a short read *r*, let *a* denote the highest-scoring alignment of *r* to a reference protein for which the functional class *c* is known. Assign *r* to the functional class *c*. For example, *c* might be an InterPro family or an eggNOG cluster. In short read analysis, each read is assigned to at most one class in any given functional classification. Many reads remain unclassified because all the reference proteins that they align to are unclassified.

A long read may contain multiple genes, and for each gene, there may be many alignments involving different taxa. To avoid redundancy in functional assignments when processing alignments between the long read and different taxa, we consider the “dominance” of individual alignments (as defined below).

Let *r* be a long read and let $a_{1},\dots,a_{k}$ be a set of DNA-to-protein alignments from *r* to a suitable protein reference sequences. Note that this set will often include alignments between the read and the same homologue in different taxa.

To reduce the number of redundant functional classes associated with *r*, we introduce the following concept. We say that an alignment *a*_*i*_*dominates* an alignment *a*_*j*_, if (1) *a*_*i*_ covers more than 50% of the read that is covered by *a*_*j*_, (2) if the bit score of *a*_*i*_ is greater than that of *a*_*j*_, and (3) both alignments lie on the same strand of *r*. Optionally, one might also require that the taxonomic identity of each protein reference sequence under consideration is compatible with the taxonomic bin assigned to the read *r*.

The set of functional classes associated with a long read *r* is then given by the functional classes associated with those alignments of *r* that are not dominated by some other alignment of *r*. Each read can be binned to all functional classes associated with it. Moreover, the set of associated classes can be used to provide simple, functional annotation of the read or contig.

To exploit that latter, we provide a dialog for exporting taxonomic and functional annotations in GFF3 format. It can be applied to any selection of taxonomic or functional classification nodes, or to a set of selected reads in the new *long read inspector*, which is described in more detail below. The user chooses a classification, and then each alignment to a reference sequence associated with that classification is exported as a CDS item. By default, only those alignments that are not dominated by another alignment are exported. In addition, the user can decide to export only those items for which the taxon associated with the corresponding reference sequence is compatible with the taxon assigned to the read.

### Reporting counts

In taxonomic or functional binning of short reads, it usually suffices to report the number of reads assigned to a specific classification node, because all reads are of a very similar length and all alignments have much the same length as the reads. For long reads or contigs, the lengths and alignment coverage can vary widely. Moreover, the number of reads contained in a contig, or contig coverage, is an additional factor to be considered. To address this, in MEGAN-LR each node can be labeled by one of the following:

1. the number of reads assigned,
2. the total length of all reads assigned,
3. the total number of aligned bases of all reads assigned, or
4. in the case of contigs, the total number of reads contained in all assigned contigs.

For long reads, by default, MEGAN–LR reports (3), the number of aligned bases, rather than (2), as this down-weights any long stretches of unaligned sequence. In addition, we use this value to determine the minimum support required for a taxon to be reported. By default, a taxon is only reported if it obtains at least 0.05*%* of all aligned bases. In MEGAN-LR, this is called the minSupport parameter. If the number of aligned bases assigned to a taxon *t* does not meet this threshold, then the assigned bases are pushed up the taxonomy until a taxon is reached that has enough aligned bases to be reported.

### Long read alignment

In this paper, we focus on taxonomic and functional binning of long reads using DNA-to-protein alignments. Currently long read sequencing technologies (Oxford Nanopore and PacBio) exhibit high rates of erroneous insertions and deletions [11, 12]. Consequently, programs such as BLASTX [22] are not suitable for such reads as they cannot handle frame-shifts.

The LAST program [23, 24] uses a frame-shift aware algorithm to align DNA to proteins and produces long protein alignments on long reads, even in the presence of many frame-shifts. Initial indexing of the NCBI–nr database (containing over 100 million sequences) by LAST takes over one day on a server. However, once completed, alignment of reads against the NCBI-nr database using the index is fast; the alignment of Nanopore reads takes roughly one hour per gigabase on a server.

The DIAMOND program [25] is widely used in microbiome analysis to compute alignments of short metagenomic reads against a protein reference database such as NCBI–nr. A new frame-shift aware alignment mode is currently under development and DIAMOND will provide an alternative to LAST in the future.

### Long read analysis

LAST produces output in a simple text–based multiple alignment format (MAF). For performance reasons, LAST processes all queries and all reference sequences in batches and alignments associated with a given query are not reported consecutively, but rather in batches.

In addition, the size of a MAF file is often very large and subsequent sorting and parsing of alignments can be time consuming. To address these issues, we have implemented a new program called “MAF2DAA” that takes MAF format as input, either as a file or piped directly from LAST, and produces a DAA (“Diamond alignment archive”) file as output [25]. The program processes the input in chunks, first filtering and compressing each chunk of data on-the-fly, and then interleaving and filtering the results into a single DAA file that contains all reads with their associated alignments. During filtering, MAF2DAA removes all alignments that are *strongly dominated* by some other alignment, to reduce a large number of redundant alignments.

In more detail, for a given read *r*, we say that an alignment *a* of *rstrongly dominates* an alignment *b* for *r*, if it covers most of *b* (by default, we require 90% coverage) and if its bit score is significantly larger (by default, we require that 0.9×bitscore(*a*)>bitscore(*b*)).

A DAA file obtained in this way can then be processed by MEGAN’s Meganizer program that performs taxonomic and functional binning, and indexing, of all reads in the DAA file. This program does not produce a new file but appends the results to the end of the DAA file, and any such “meganized” DAA file can be directly opened in MEGAN for interactive analysis. We have modified MEGAN so that it supports frame-shift containing alignments. The final DAA file is usually around ten times smaller than the MAF file produced by LAST.

### Long read visualization

Interactive analysis tools for short read microbiome sequencing data usually focus on representing the taxonomic and functional classifications systems used for binning or profiling the reads, for example reporting the number of reads assigned to each class. In addition, some tools provide a reference-centric visualization that displays how the reads align against a given reference sequence. However, visualizations of the short reads themselves are usually not provided.

**Fig. 2:**
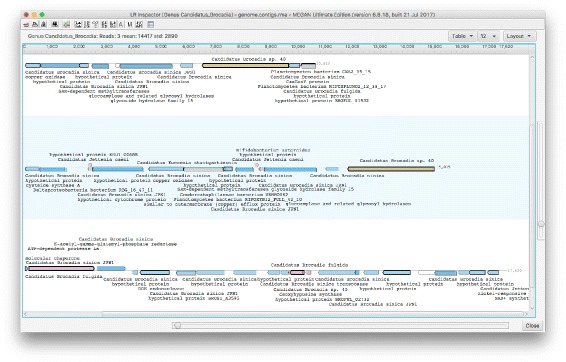
This screen shot of the MEGAN-LR long read inspector shows three contigs assigned to the genus *Candidatus Brocadia*, with alignments to more specific taxa. Alignments to reference protein sequences are shown as arrows, colored by species of the references; blue for *Candidatus Brocadia sinica*, brown for *Candidatus Brocadia sp. 40* and pink for *Candidatus Brocadia fulgida*. Alignments are labeled by taxonomic and functional classes associated with the corresponding reference proteins

For long read or contigs, there is a need for visualization techniques that make it easy to explore the taxonomic and functional identity of reference sequences to which the reads align. To address this, we have designed and implemented a *long read inspector* (using JavaFX) that allows one to investigate all long reads assigned to a given taxonomic or functional class (see Fig. 2).

In this tool, each long read or contig *r* is represented by a horizontal line and all corresponding aligned reference sequences are shown as arrows above (forward strand alignments) or below (reverse strand alignments) the line. The user can select which annotations to display in the view. For example, if the user requests Taxonomy and InterPro annotations, then all reference sequences will be labeled by the associated taxonomic and InterPro classes. The user can search for functional attributes in all loaded reads.

Let *a* be an arrow representing an alignment of *r* to a reference sequence associated with taxon *s*. We use a hierarchical coloring scheme to color such arrows. Initially, we implicitly assign a color index to each taxon, e.g., using the hash code of the taxon name. For each arrow *a* with associated reference taxon *s* we distinguish between three different cases. First, if *s*=*t*, then we use the color assigned to *t* to color *a*. Second, if *s* is a descendant of *t*, then *t* has a unique child *u* that lies on the path from *t* down to *s* and we use the color of *u* to color *a*. Otherwise, we color *a* gray to indicate that the taxon associated with *a* is either less specific or incompatible with *t*.

For example, if a read *r* is assigned to the genus *Candidatus Brocadia* and has an alignment to the strain *Candidatus Brocadia sinica JPN1*, then we color the corresponding arrow *a* using the color that represents the species *Candidatus Brocadia sinica*.

This is a useful strategy when used in combination with the taxonomic binning procedure described above: a read *r* is binned to the lowest taxon *t* that covers 80% (by default) of the aligned read and the taxonomy-based coloring makes it easy to see how the different taxonomic classes below *t* contribute. For example, if all arrows on one half of the read have one color and all arrows on the other half have some other color, then this may indicate a chimeric read or misassembled contig.

**Fig. 3:**
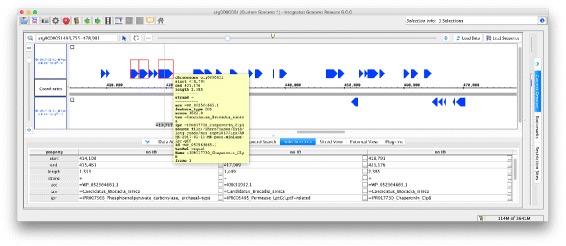
Example of long read data exported from MEGAN-LR and imported into the IGB genome browser [26]

As discussed above, an alternative approach is to export reads and their alignments in GFF3 format and then to use a genome browser such as IGB [26] to explore them (see Fig. 3).

### LAST+MEGAN-LR

**Fig. 4:**
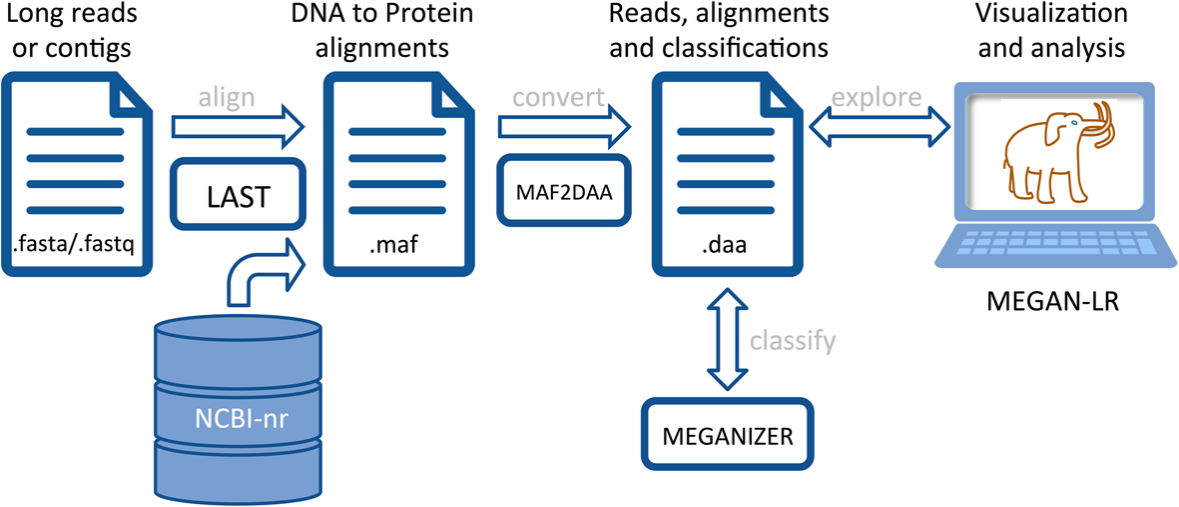
The LAST+MEGAN-LR pipeline. Long reads or contigs are aligned against the NCBI-nr database using LAST and the resulting MAF file (multiple alignment format) is converted to DAA format (Diamond alignment format), including filtering of dominated alignments. Taxonomic and functional binning of the reads or contigs is then performed using the Meganizer program and the results are appended to the DAA file. The meganized DAA file can then opened and interactively analyzed in MEGAN-LR
- Either pipe the output of LAST directly to MAF2DAA, or apply MAF2DAA to the MAF file generated by LAST, to obtain a much smaller output file in DAA format.
- Meganize the DAA file either using the Meganizer command-line tool or interactively in MEGAN.
- Open the meganized DAA file in MEGAN for interactive exploration using the long-read inspector. Export annotated reads in GFF3 format for further investigation, e.g. using a genome browser such as IGB [26] or Artemis [27].

In summary, we propose to use the following pipeline to analyze metagenomic long reads and contigs (see Fig. 4):

- Align all reads against a protein reference database (such as NCBI-nr) using LAST, producing MAF output.

### Nanopore sequencing

To obtain a Nanopore dataset, we sequenced the genomic DNA of the Microbial Mock Community B (even, high concentration, catalog nr. HM-276D, BEI Resources). Library preparation was performed using a Low Input by PCR Genomic Sequencing Kit SQK-MAP006 (Oxford Nanopore Technologies, Oxford, UK) for 2D sequencing. Briefly, 100 ng of genomic DNA was sheared in a Covaris g-TUBE (Covaris, Inc., Woburn, MA, USA) at 6000 rpm, treated with PreCR (New England Biolabs, Ipswich, MA, USA) and used as input for adapter ligation according to the ONT protocol. Adapter-ligated DNA was further amplified with the LongAmp Taq 2X Master Mix (NEB) using the following program: 95°C 3 min; 18 cycles of 95°C 15 sec, 62°C 15 sec, 65°C 10 min; 65°C 20 min. Sequencing was performed using an early access MinION device (ONT) on a FLO-MAP003 flowcell (ONT). Raw fast5 files were obtained with MinKNOW (v0.50.2.15, ONT) using a 48 h genomic sequencing protocol, basecalled with ONT’s proprietary Metrichor cloud-based basecalling service and the 2D Basecalling for SQK-MAP006 v1.34 workflow.

Genomic DNA from the lab scale Anammox enrichment reactor described in Liu et al. [28] was extracted using the FastDNA SPIN Kit for Soil with 4x homogenization on the FastPrep instrument (MP Bio). The DNA was further purified using Genomic DNA Clean and Concentrator -10 Kit (Zymo Research). Approximately 1700 ng of extracted DNA was used for library preparation using a Ligation Sequencing Kit SQK-LSK108 (Oxford Nanopore Technologies, Oxford, UK) for 1D sequencing according to the manufacturer protocol. Sequencing was performed using an early access MinION device (ONT) on a SpotON FLO-MIN106 flowcell (R9.4). The run was stopped after 22 h due to low number of active pores. Fast5 files were obtained with MinKNOW (v1.3.30, ONT) using a 48 h genomic sequencing protocol. Basecalling was performed using Metrichor (Instance ID:135935, 1D Basecalling for FLO-MIN106 450 bps_RNN (rev.1.121)).

### Parameters

The MEGAN-LR approach employs a number of different user-specified parameters. The main effect of changing any of these is usually a shift in the trade-off between false positive and false negative taxonomic assignments. What balance of false positives and false negatives is ideal depends on the biological question at hand, and so the parameters may have to be adjusted by the user.

The minSupport parameter (default setting 0.05*%*) sets the “level of detection”, that is, it is used to decide whether a taxonomic node has been assigned enough weight (such as number of reads or number of aligned bases, say) so as to appear in the displayed tree. If the threshold is not met, then the weights are pushed up the tree until enough weight has been accumulated. Lowering this threshold will improve sensitivity for low-abundance species while increasing the risk of false positives induced by the erroneous assignment of individual reads, i.e., due to random hits or database errors. Increasing this threshold will decrease false positives while causing more low-abundance taxa to be missed.

The topPercent parameter (default value 10%) is used to determine which alignments on the same interval of a read are considered significant. An alignment is only considered significant if its bitscore lies within the given percentage of the bitscore for the best alignment. Setting this threshold too small will result in false positive assignments based on chance differences in alignment score, whereas setting this threshold too large will result in false negatives on lower taxonomic ranks due to assignment to higher taxonomic classes.

The percentToCover parameter (default value 80%) influences at what rank of the taxonomy a long read will be placed. Setting this parameter too high or too low will usually result in less specific assignments.

LAST alignment of long reads against the NCBI-nr database can produce very large files due to large numbers of alignments covering the same segment of reads. The concept of strong-domination was developed to address this issue. By default, MEGAN-LR uses a setting of MinPercentCoverToStronglyDominate = 90% and TopPercentScoreToStronglyDominate=90% to filter reads.

When reporting functional classes of intervals of a long read, a key problem is which alignments to report on. In practice, using all alignments found for a read produces too many redundant gene calls. Here MEGAN-LR uses a parameter MinPercentCoverToDominate = 50% to filter the alignments that are reported.

In the “Results” section, we illustrate the effect of varying most of these parameters on the performance of MEGAN-LR on mock community data.

## Simulation study

To evaluate the performance of the proposed LAST+MEGAN-LR approach and, in particular, of the interval-union LCA algorithm, we undertook a simulation study to estimate the sensitivity and precision of the algorithm, following the protocol reported in [15], as defined below. We attempted to model two major obstacles in metagenomic studies, namely sequencing errors and the incompleteness of reference databases.

Our simulation study is based on a set *P* of 4282 prokaryotic genomes from NCBI for which both annotated genomes and annotated sets of proteins are available, downloaded in March 2017. In addition, we identified a subset *Q* of 1151 genomes that consists of all those organisms in *P* whose genus contains at least 2 and at most 10 organisms in *P*, and for which a full taxonomic classification is given. Note that *Q* can be partitioned into nine different categories, based on the number 2−10 of organisms in *Q* that the corresponding genus contains.

For each target species *t* in *Q*, we performed the following “leave-one-out” evaluation:

- First, we collected a set of *R* of 2000 simulated reads from the genome sequence of *t* using NanoSim [29], a read simulator that produces synthetic reads that reflect the characteristic base-calling errors of ONT reads, running in linear mode.
- Second, we constructed a protein reference database $D_{\hat {t}}$ that contained all proteins associated with all organisms in *P* except for *t* (“leave one out”).
- Third, we performed taxonomic binning of all reads in *R* using LAST+MEGAN-LR as follows. We first build a LAST reference index on $D_{\hat {t}}$, then aligned all reads in *R* against $D_{\hat {t}}$ using LAST, with a frameshift cost of 15, and then performed taxonomic binning of all reads in MEGAN using the interval-union LCA algorithm (default parameters).
- Fourth, for comparison, we also ran the taxonomic binning program Kaiju[15] on *R* and $D_{\hat {t}}$, building a custom Kaiju index on $D_{\hat {t}}$. We performed taxonomic binning of simulated reads using Kaiju’s greedy mode, with the maximum number of allowed substitutions set to 5.

To be precise, we ran each of the four steps twice to produce two simulation datasets, each containing 2,000 reads per target species. The first dataset was produced using the ecoli_R73_2D (R7.3) simulator profile, whereas the second was produced using the ecoli_R9_2D (R9) profile. Both profiles were downloaded from the NanoSim FTP address (http://ftp.bcgsc.ca/supplementary/NanoSim/) in April 2017. The R7.3 profile introduces more errors in reads and should make it harder for analysis methods to identify appropriate reference sequences.

To compare the performance of MEGAN-LR and Kaiju, we calculated the sensitivity and precision of taxonomic assignments at the genus, family and order levels. In more detail, following the approach used in [15], we define *sensitivity* as the percentage of reads in *R* that are assigned either to the correct taxon or to one of its descendants. We define *precision* as the percentage of reads that are assigned correctly, out of all reads that were binned to any node that is not an ancestor of the correct taxon.

## Results

We have implemented the interval-union LCA algorithm and the modified functional binning algorithm. In addition, we have implemented a new long read interactive viewer. We provide methods for exporting long read annotations in GFF3 format. Our code has been integrated into the open source edition of MEGAN. In addition, we have modified MEGAN (and all tools bundled with MEGAN) so as to support DNA-to-protein alignments that contain frame-shifts. We use the term MEGAN-LR (MEGAN long read) to refer to this major extension of MEGAN.

### Simulation study

**Fig. 5:**
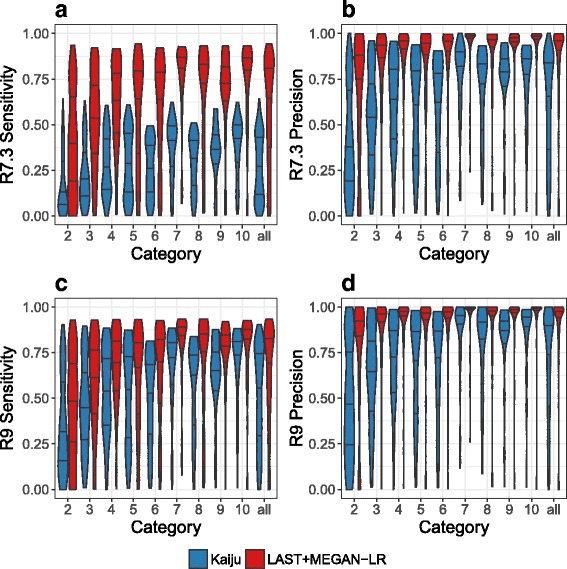
Violin plots comparing the performance of LAST+MEGAN-LR and Kaiju for two simulation studies, one based on a R7.3 Nanopore chemistry profile and the other based on a R9 Nanopore chemistry profile. In both cases, we report the sensitivity (percentage of reads assigned to the correct taxon) and precision (percentage of reads assigned correctly out of all reads not binned to an ancestor of the correct taxon) of taxonomic assignments. This is done at the genus level for nine different categories of genera (reflecting the number of species in the genus from which the target species was removed), and for all. Results for the R7.3 profile are shown in **a** and **b**, and results for the R9 profile are shown in **c** and **d**

The results of our simulation study are shown in Fig. 5, where we summarize the sensitivity and precision scores achieved at genus level by LAST+MEGAN-LR and Kaiju, for both the R7.3 and R9 datasets. In all cases, LAST+MEGAN-LR shows better sensitivity and precision than Kaiju. As expected, both methods are less sensitive on the R7.3 data, as many reads remain unclassified. However, the difference in performance between the two methods is larger on the R7.3 data, and we suspect that this is due to the ability of LAST to perform frame-shift aware alignments and thus to accommodate erroneous insertions and deletions.

**Fig. 6:**
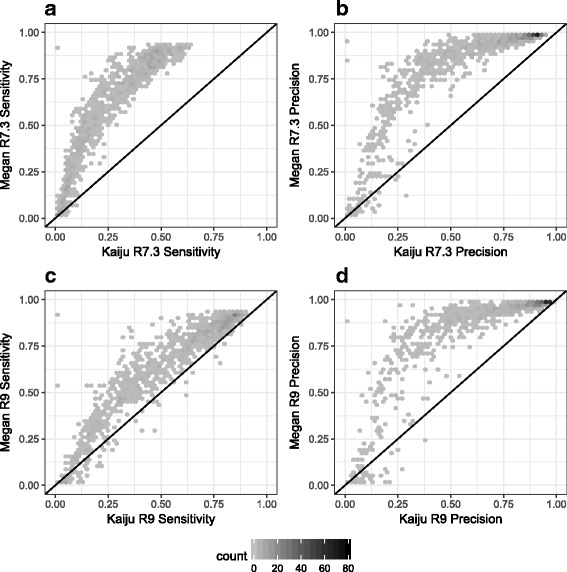
Here we plot the sensitivity and precision at genus level for Kaiju versus LAST+MEGAN-LR on the R7.3 samples in **a** and **b**, and on the R9 samples in **c** and **d**, respectively

Per-dataset performance analysis of LAST+MEGAN-LR and Kaiju is presented in Fig. 6. This shows that LAST+MEGAN-LR outperforms Kajiu on a vast majority of the simulated datasets, with Kajiu sometimes showing better performance when the sensitivity or precision is very low.

Kaiju is many times faster than LAST+MEGAN-LR. However, the latter approach computes and uses all relevant protein alignments, and these are also used to perform functional analysis of the reads or contigs. Hence, we suggest to use Kaiju to obtain a fast, first taxonomic profile for a set of long reads or contigs, and then to use LAST+MEGAN-LR to perform a more accurate and detailed subsequent analysis.

### PacBio reads on HMP mock community

To test LAST+MEGAN-LR on a publicly available PacBio mock community dataset, we downloaded “HMP dataset 7” from the PacBio website https://github.com/PacificBiosciences/DevNet/wiki/Human_Microbiome_Project_MockB_Shotgun in April 2017. This dataset contains 319,703 reads of average length 4,681 bp. It was sequenced using the P5 polymerase and C3 chemistry.

LAST alignment against the NCBI-nr database (downloaded January 2017) resulted in protein alignments for 284,728 reads (89% of all reads). MEGAN-LR analysis using the interval-union LCA algorithm assigned 1054 megabases (Mb) aligned bases to taxonomic nodes. Of these, 945.3 Mb were assigned to bacterial genera, with no false positives. A total of 758.4 Mb of aligned sequences were assigned to bacterial species, of which 755 Mb were assigned to true positive species (that is, species known to be contained in the mock-community), whereas approximately 3.4 Mb (0.4*%*) were assigned to false positive species. The 20 bacterial species in the mock community received between 2.8 Mb (0.37*%*) and 145 Mb (19%) aligned bases assigned at the species level, whereas the highest false positive species obtained 1.1 Mb (0.14*%*).

Kaiju classified 280,465 of these reads, assigning 128,774 to a species or lower rank node with a true positive rate of 76.9*%*. 209,435 reads were assigned to a genus or lower rank node with a true positive rate of 84.5*%*.

To investigate the use of LAST+MEGAN-LR on assembled reads, we assembled this set of reads using minimap (options -Sw5 -L100 -m0 -t8) and miniasm (version 0.2, default options) [30] and obtained 1130 contigs, with a mean length of 43,976 and maximum length of 1,272,994. LAST alignment against the NCBI-nr database resulted in 41.8 Mb of aligned sequences. Of this, 41.1 Mb and 38.6 Mb, were assigned to bacterial genus and species nodes, respectively, with no false positives and only one false negative species.

### PacBio reads on Singer et al. mock community

Our analysis of PacBio reads recently published on a mock-community containing 26 bacterial and archaeal species [31] gave rise to results of similar quality. Of 53,654 reads of average length 1,041 and maximum length 16,403, exactly 51,577 received LAST alignments against NCBI-nr. Of 49.5 Mb of aligned sequences, 45.8 Mb were assigned to prokaryotic genera, with no assignments to false positive species. The amount of sequence assigned at the species level was 36.8 Mb, all of which was assigned to true positive species.

Of the 26 species in the mock community, two are not reported in the analysis and therefore constitute false negative species. These make up approximately 0.01*%* (*Nocardiopsis dassonvillei*) and 0.1*%* (*Salmonella bongori*) of the community and are thus on the borderline of detection using the default settings of MEGAN-LR. By default, MEGAN-LR requires that a taxon receives at least 0.05*%* of all aligned bases before it is reported.

On this data, Kaiju assigned 47,056 reads at the species level, with a true positive rate of 98.7*%*.

### Nanopore reads on HMP mock community

To perform the first test of our new methods on Nanopore data, we sequenced the content of the Genomic DNA from Microbial Mock Community B, as described in the “Methods” section. We obtained 124,911 pass reads of average length 2870, including all template-, complement- and 2D reads.

The LAST alignment against the NCBI-nr database resulted in protein alignments for 57,026 reads (45.6*%* of all reads). MEGAN-LR analysis assigned a total of 110 Mb aligned bases. Of these, 100 Mb were assigned to bacterial genera, with a false positive assignment rate of 0.1*%*. Approximately 71.9 Mb of aligned sequences were assigned at the species level, with a false positive rate of 0.9*%*. The 20 bacterial species in the mock community received between 0.36 Mb (0.5*%*) and 12.2 Mb (17%) aligned bases assigned at the species level, whereas the highest false positive species obtained 0.21 Mb (0.3*%*). Around 66 kb of all aligned sequences (0.05*%*) were falsely assigned to Eukaryota.

Kaiju exhibited a higher false positive rate than LAST+MEGAN-LR on these Nanopore reads, namely 19.8*%* and 12.6*%* at the species and genus level, respectively. The program assigned 22,433 reads at the species level and 39,173 reads at the genus level.

### Application to anammox data

To illustrate the utility of our new methods in a research context, we applied Nanopore sequencing to a sample obtained from a laboratory bio-reactor enriched for anaerobic ammonium oxidizing bacteria (AnAOB) [32], as described in the “Methods” section. We obtained 71,411 reads of average length 4658 and maximum length 30,846.

LAST alignment against the NCBI-nr database resulted in protein alignments for 64,097 reads (90% of all reads). MEGAN-LR analysis assigned a total of 212 Mb aligned bases. Of these, 94 Mb were assigned to bacterial genera and 112 Mb to bacterial species. The reason why there are more assignments to species than there are to genera is that some of the species present do not have a genus designation in the NCBI taxonomy. The top ten bacterial species assignments are shown in Table 1. This indicates that the most abundant organism in the sample is *Candidatus Brocadia sinica*, a known AnAOB species.

**Table 1:** The ten top bacterial species identified in a Nanopore dataset taken from an anammox enrichment bioreactor, by the number of bases aligned to corresponding reference proteins

Species	Aligned (Mb)
*Candidatus Brocadia sinica*	84.9
*Armatimonadetes bacterium OLB18*	8.8
*Bacteroidetes bacterium OLB12*	4.8
*Rhodocyclaceae bacterium UTPRO2*	2.9
*Chloroflexi bacterium OLB13*	2.7
*Nitrospira sp. OLB3*	1.5
*Streptomyces sp. SolWspMP-5a-2*	1.1
*Anaerolineae bacterium UTCFX5*	0.6
*Pseudorhodoplanes sinuspersici*	0.4

For *Candidatus Brocadia sinica*, this suggests at least ten-fold coverage of the genome

**Fig. 7:**
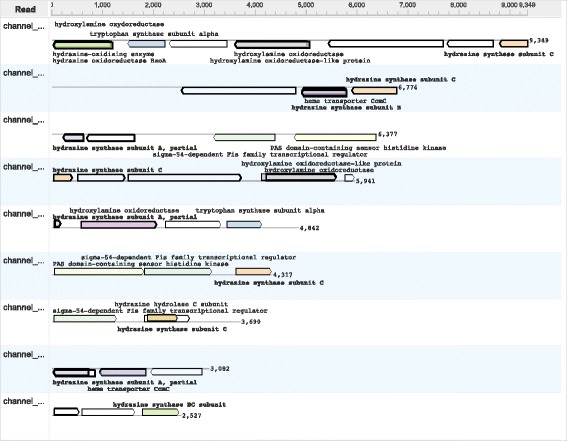
Long read inspector showing nine reads in the anammox sample that together contain all three subunits of the hydrazine synthase gene, labeled hydrazine synthase subunit A, partial, hydrazine synthase subunit B and hydrazine synthase subunit C

Functional binning in MEGAN-LR allows one to summarize counts at different levels of detail. For example, in Table 2 we list the number of alignments to genes for the main KEGG categories of metabolism. MEGAN-LR also makes it possible to investigate function in detail. For example, the anammox process relies on the extremely reactive intermediate hydrazine, produced by the enzyme hydrazine synthase, comprised of the three protein subunits HSZ- *α*, HZS- *β* and HZS- *γ* [33]. Using MEGAN-LR, we identified eight reads that together contain all three subunits, see Fig. 7.

**Table 2:** For each of the main KEGG categories of metabolism, we report the number of alignments against KEGG Orthology reference sequences for the given category, and the number of different KEGG Orthology groups (KOs) involved in such alignments

KEGG metabolism categories	# Alignments	# KOs
Carbohydrate metabolism	9691	347
Amino acid metabolism	8519	371
Energy metabolism	4909	225
Metabolism of cofactors and vitamins	2826	197
Nucleotide metabolism	2675	124
Lipid metabolism	2564	95
Xenobiotics biodegradation and metabolism	1738	116
Glycan biosynthesis and metabolism	1684	114
Metabolism of other amino acids	1344	73
Metabolism of terpenoids and polyketides	1156	63
Biosynthesis of other secondary metabolites	1076	54

These results are based on a LAST+MEGAN-LR analysis of Nanopore reads from an anammox enrichment bioreactor

To illustrate the use of LAST+MEGAN-LR on assembled reads, we assembled this set of reads using minimap (options -Sw5 -L100 -m0 -t8) and miniasm (default options) [30] and obtained 31 contigs, with a mean length of 129,601 and maximum length of 750,799. LAST alignment against the NCBI-nr database resulted in 2.98 Mb of aligned sequences. The interval-union LCA algorithm assigned 13 contigs and 96% of all aligned bases to *Candidatus Brocadia sinica*.

### Performance

To illustrate the computational resources required by the LAST+MEGAN-LR approach, we measured the wall-clock time and memory consumption on the four datasets discussed above. In addition, we considered a further unpublished Nanopore dataset obtained from cheese, consisting of 34 million reads of average length 1460 and maximum length 229,439 (unpublished data provided by the Dutton Lab, UCSD, during the Santa Barbara Advanced School of Quantitative Biology 2017). The programs were run on a Linux server with 32 cores and 512 GB of main memory.

We ran LAST using a volume size setting (parameter -s) of 20 GB (the maximum value), and recorded the peak memory used by the program. We set the maximum memory limit of MEGAN to between 5 GB and 10 GB, depending on the input size. We summarize our measurements in Table 3. The LAST alignment of reads was performed against the entire NCBI-nr protein database and the total size of the LAST index was 215 GB. This step took between a few minutes and a few hours, depending on the size of the input file. The subsequent two steps of conversion and meganization took less than half as long as alignment. By using a smaller LAST volume size, the whole pipeline can also be run on a computer with 16 GB main memory, such as a laptop.

**Table 3:** Performance of the LAST+MEGAN-LR pipeline

Step	Input	Output	Runtime	Memory
PacBio reads on HMP mock community				
Align	Reads file (1.5 GB)	MAF file	119 min	23 GB
Convert	MAF file (49 GB)	DAA file	29 min	5 GB
Classify	DAA file (4.2 GB)	Meganized DAA file (4.5 GB)	6 min	5 GB
PacBio reads on Singer et al. mock community				
Align	Reads file (56 MB)	MAF file	10 min	22 GB
Convert	MAF file (8.9 GB)	DAA file	5 min	5 GB
Classify	DAA file (197 MB)	Meganized DAA file (415 MB)	1 min	5 GB
Nanopore reads on HMP mock community				
Align	Reads file (191 MB)	MAF file	10 min	22 GB
Convert	MAF file (6.1 GB)	DAA file	3 min	5 GB
Classify	DAA file (553 MB)	Meganized DAA file (644 MB)	1 min	5 GB
Anammox data				
Align	Reads file (336 MB)	MAF file	31 min	24 GB
Convert	MAF file (8.5 GB)	DAA file	4 min	5 GB
Classify	DAA file (371 MB)	Meganized DAA file (500 MB)	2 min	5 GB
Cheese data				
Align	Reads file (5.1 GB)	MAF file	251 min	24 GB
Convert	MAF file (93 GB)	DAA file	90 min	10 GB
Classify	DAA file (3.1 GB)	Meganized DAA file (3.5 GB)	5 min	10 GB

For each of five long read datasets, we report the wall-clock time and main memory required by LAST to align against the NCBI-nr database, for MEGAN to convert the LAST MAF output files into DAA format, and then for MEGAN to classify the reads so as to meganize the DAA file, respectively. The computations were performed on a Linux server with 32 cores and 512GB memory

### Parameters

To investigate the effect of setting particular parameter values, we analyzed the three mock communities employing a range of different values for minSupport, topPercent and percentToCover. We used the values 0, 0.025, 0.05, 0.075 and 0.1 for minSupport; 0, 5, 10 and 20 for topPercent; and 50, 60, 70, 80, 90 and 100 for percentToCover, respectively. Starting with the DAA file containing the LAST alignments of the reads against NBCI-nr, we ran the classification step of the MEGAN-LR pipeline on all possible combinations of values for the three parameters, with all other parameters set to their default values. We turned off the strong-domination filter for the cases in which topPercent equals 20, because that filter removes any alignment whose score lies 10% below that of the best overlapping hit.

**Fig. 8:**
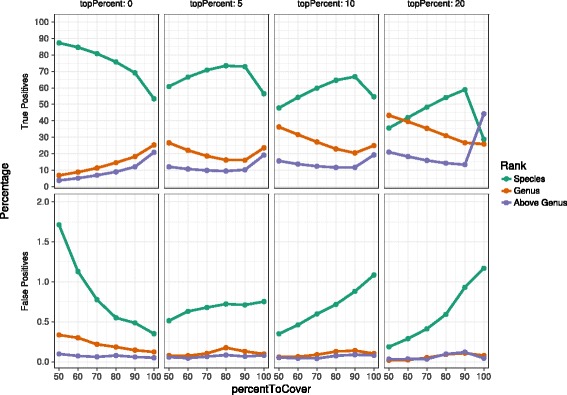
The effect of changing the topPercent and percentToCover parameters for the analysis of the Nanopore HMP mock community. True positive and false positive rates are reported for each combination of parameters at the levels of species and genus, and for the sum of ranks above genus. The rate is calculated as the number of correctly assigned bases divided by the total number of bases assigned at the respective taxonomic level

For all combinations of parameters, we calculated the rate of true positives and false positives for the number of assigned bases at the species and genus ranks, as well as for the number of assigned bases at any rank above genus. Figure 8 shows these values for Nanopore reads on HMP mock community. The figures for PacBio reads on the HMP and the Singer et al. mock community are available in the supplementary material. We also decided to omit the minSupport parameter in the figures as it showed little to no variability for any value above 0. Turning off minSupport causes spurious assignments of some reads (up to 4% at species level).

As depicted in Fig. 8, increasing the percentToCover parameter improves the specificity of the true positive assignments (i.e. more reads are binned at lower ranks), but also increases the rate of false positives.

Using a higher value of the topPercent parameter results in more alignments being considered by the LCA algorithm and thus results in a more conservative or less specific binning of reads.

We would like to emphasize that the datasets tested for the effects of parameters in this study are mock communities of species whose proteins are well represented in the reference database. While Fig. 8 suggests setting TopPercent to 5 % and percentToCover to 90 %, we suggest that in practice both values should be relaxed slightly, to 10 and 80 %, respectively, so as to account for the fact that environmental microbes are usually not so well represented by reference sequences.

## Discussion

The application of long read sequencing technologies to microbiome samples promises to provide a much more informative description of the genetic content of environmental samples. The alignment of long reads against a protein reference database is a key step in the functional analysis of such data. Here we show that such protein alignments can also be used to perform accurate taxonomic binning using the interval-union LCA algorithm.

Our simulation study suggests that LAST+MEGAN-LR performs taxonomic binning more accurately than Kaiju. The reported results on mock community datasets indicate a high level of accuracy down to the species level when the corresponding species are represented in the protein reference database. In addition, the computed protein alignments can be used to identify genes and MEGAN-LR provides a useful visualization of the annotated sequences.

The main motivation for developing these new methods is to assist our work on the study of microbial communities in enrichment bio-rectors, where long read sequencing promises to provide access to near-complete genome sequences of the dominating species.

The simple assembly of the anammox data presented in this paper places the dominant species into 11 contigs of length greater than 100 kb, containing about 2.8 Mb of aligned sequence and 3.7 Mb of total sequence. This suggests that a more careful assembly, assisted by a set of high quality MiSeq reads, should result in a nearly complete genome.

Our simulation study did not incorporate chimerism or similar artifacts. Because Kaiju uses a heuristic based on the longest match found, we suspect that Kaiju will perform poorly on chimeric reads or misassembled contigs, assigning such a read to one of the source taxa. In contrast, the interval-union LCA algorithm requires by default that 80% of the aligned read is assigned to a taxon and so in practice, such reads will often be placed on a higher taxonomic node.

All datasets discussed in this paper are available here: http://ab.inf.uni-tuebingen.de/software/downloads/megan-lr.

## Conclusions

There is increasing interest in using long reads in microbiome sequencing and there is a need to adapt short read tools to long read datasets. In this paper we present an extension of the widely-used metagenomic analysis software MEGAN to long reads. With MEGAN-LR, we provide new algorithms for taxonomic binning, functional annotation and easy interactive exploration of metagenomic long reads and contigs, based on DNA-to-protein alignments. Our work suggests that the presented LAST+MEGAN-LR pipeline is sufficiently fast and accurate.

## Reviewers’ comments

### Reviewer’s report 1: Nicola Segata and Moreno Zolfo

Reviewer’s comments: The authors present here a novel computational pipeline to address the issue of taxonomical and functional classification of long reads. The authors correctly underline that long reads from emerging sequencing technologies are currently a computational challenge in the field of metagenomics. Indeed, not much attention has been dedicated to the taxonomic identification of long reads, and the author developed an extension of the previously published MEGAN software, which they call MEGAN-LR. The pipeline works with long nucleotide reads which are mapped against a protein database using LAST, it accounts for read that align against more than one protein, and is frameshift aware. The authors provide convincing evidences on the accuracy and precision of MEGAN-LR on synthetic data and mock communities sequenced ad-hoc. This review was performed by Nicola Segata and Moreno Zolfo

As summarized in my comments above, I think this is a well written and clear paper. I do not think there are many major issues, but there are several points that the authors should at least consider addressing to improve the paper:

1. It would be useful for the general comprehension of the frameset in which MEGAN-LR is set, to understand why the authors decided to focus on protein-based taxonomic assignment. Most of the other existing algorithms use nucleotide-based approaches. I would suggest to add a paragraph exploring the advantages and disadvantages of the two approaches.
**Author’s response:** *We have added a paragraph discussing this to the* Background *section.*
2. The default threshold to report the presence for a taxon is set to 0.05% of the total aligning bases. Since the overall performance of the algorithm could be dramatically affected by this parameter, it would be nice to see how the precision and specificity of MEGAN- LR vary when changing the threshold. Also, I think that the authors should clarify on how this threshold was chosen as default: was it the result of a parameter- optimization of some sort?
  **Author’s response:***We have added a section on* “Parameters” *to* Methods.
3. Similarly, one could test the impact of the threshold that is used to determine whether a LAST alignment is strongly dominated by another alignment. Since this value is set by default to 90%, it would be interesting to see the behaviour of the mapper at different thresholds.
  **Author’s response:***We have added a section on* “Parameters” *to* Methods.
4. The fact that some alignments in the MAF file are eliminated if they are strongly dominated by another alignment can affect the correct placement of a read. How did the authors decide the default thresholds by which this mechanism is implemented in MEGAN-LR?
  **Author’s response:***We have added a section on* “Parameters” *to* Methods.
5. Overall, a precise estimate on the memory and CPU requirements of MEGAN-LR is not provided. I think this point should be reported more clearly, by providing the computational resources used by MEGAN-LR in the analysis. Specifically, I think it would be useful to report how much CPU time and memory were required in each of the validations step. Moreover, it would be also useful to have an estimate on the order of magnitude of time required to analyse a whole average PacBio/Nanopore metagenome.
  **Author’s response:***We have added a section on* “Performance” *to* Results.
6. Figure 5, the performances of Kaiju and LAST+MEGAN-LR are binned by the number of species in the genus. It would be interesting to see in the same box plot also the summed (i.e. overall) distributions for each subplot.
**Author’s response:** *To each subplot, we have added a category that summarizes all datasets.*
7. The comparison between Kaiju and MEGAN-LR is performed only on the simulated dataset. I would suggest to run Kaiju also on the PacBio and Nanopore reads from the mock communities, if the genomes of the species present in the communities are available and well annotated. This should provide further support to the higher specificity and precision of MEGAN-LR.
**Author’s response:** *We have added true positive and false positive rates of Kaiju’s assignments for mock communities against NCBI-nr to their respective sections.*
8. Another computational tool that is addressing the problem of long-reads mapping is MinHash (Jain et al., 10.1101/103812). It is understandable that the validation was conducted only on Kaiju (as it is the only tool using protein-alignments). Nevertheless, it would be interesting to see the other approaches compared.
**Author’s response:** *A comparison against DNA-based analysis approaches is beyond the scope of this paper.*
9. There is no much on the task of “functional classification” in the “Results” section. Estimating the functional potential of a microbiome is an important task, and it would be very nice if the authors provide some details, validation, and application on real data for this. ror example could the authors provide some comments on the functional landscape detectable with MEGAN-LR of the anammox dataset?
**Author’s response:** *We have added a high-level summary genes assigned to KEGG metabolic categories and also a detailed inspection of the key hydrazine syntase subunits for the anammox sample.*

### Reviewer’s report 2: Pete James Lockhart

Reviewer’s comments: The manuscript by Huson et al. describes and evaluates a novel approach for analyzing long sequence reads and these to taxa and functional categories. The approach will be welcomed by biologists as it provides objective criteria and an interactive means to evaluate the taxonomic identity of species in metagenomics samples.

Identify genome functional characteristics. The latter will include e.g. virulence and pathogenicity, and provides a means e.g. for assessing health risk posed by micro- organisms in metagenomics samples. I have indicated some minor points of communication that should be considered.

1. Also a number of default thresholds are indicated for different stages of analysis, e.g. 80% threshold for the LCA assignment, 50% for the alignment dominance criterion, 0.05% for MEGAN-LR reporting. It would help potential users to have more insight into the thinking behind these values, and whether or not additional threshold values should be considered.
  **Author’s response:***We have added a section on* “Parameters” *to* Methods.

### Reviewer’s report 3: Serghei Mangul

Reviewer’s comments:

1. The authors propose protein based alignment. Is there an advantage to use protein-based alignment versus nucleotide-based alignment?
**Author’s response:** *We have added a paragraph discussing this to the Background section.*
2. The nucleotide- based methods (for example Centrifuge) have been excluded from the comparison. Including those methods (by using the comparable database with nucleotide sequences) can be valuable. Also, this will provide a general comparison of nucleotide-based versus protein based performance of metagenomic tools.
**Author’s response:** *While we agree that such a comparison would be useful, such a comparison against DNA-based analysis approaches is beyond the scope of this paper.*
3. p.9, line 46. More information about the leave-one-out experiment is required. What is the motivation for the experiment? Does it refer to removing one reference genome, from which reads were simulated? Such experiment can quantify, the possibility of misassignment of reads to the close-related genome, due to the incompleteness of the reference.
**Author’s response:** *Yes, all genes associate with the source genome are removed from the reference database.*
4. p.10, line 18. What is the maximum number of mismatches allowed by MEGAN-LR? The effect of this parameter on the performance of both Megan-LR and Kaiju needs to be explored.
**Author’s response:** *While the number of mismatches is an important parameter for DNA-DNA alignments, it does not usually play a role in amino-acid alignments.*
5. p.10. How was the performance on the species level?
**Author’s response:** *Our study follows the one published in the Kaiju paper and does not allow an assessment of species-level performance due to its ‘leave one species out” approach.*
6. p.10. The paper report sensitivity and precision on the read level. It would be interesting to know such performance on different taxa levels. In such, case sensitivity, for example, would be the percentage of taxa correctly identified.
**Author’s response:** *We have added supplementary plots for higher taxonomic levels to the companion website.*
7. p.11. The contribution of LAST algorithms to the superiority of MEGAN-LR in comparison to other methods needs to be quantified. One way to do so is to compare the performance of Kaiju with LAST instead of current alignment algorithm.
**Author’s response:** *As an aligner, LAST does not perform taxonomic binning and so a comparison of Kaiju with LAST without MEGAN-LR is not possible.*
8. p.12, line 24. A more extensive analysis is required. Besides, FN species, it will be interesting to know the number of TP, FP and general sensitivity and precision of each taxonomic level.
  **Author’s response:***FN levels are very low for the mock data. We now report TP and FP in* Fig. 8.

## Abbreviations

MEGAN-LR: long read extension of the metagenome analysis tool MEGAN
